# Detection of Multi-Layered Bond Delamination Defects Based on Full Waveform Inversion

**DOI:** 10.3390/s24124017

**Published:** 2024-06-20

**Authors:** Jiawei Wen, Can Jiang, Hao Chen

**Affiliations:** 1 Institute of Acoustics, Chinese Academy of Sciences, Beijing 100190, China; wenjiawei@mail.ioa.ac.cn; 2 School of Electronic, Electrical and Communication Engineering, University of Chinese Academy of Sciences, Beijing 100049, China

**Keywords:** ultrasonic guided waves, plate structures, full waveform inversion, delamination defects

## Abstract

This study aimed to address the challenges encountered in traditional bulk wave delamination detection methods characterized by low detection efficiency. Additionally, the limitations of guided wave delamination detection methods were addressed, particularly those utilizing reflected waves, which are susceptible to edge reflections, thus complicating effective defect extraction. Leveraging the full waveform inversion algorithm, an innovative approach was established for detecting delamination defects in multi-layered structures using ultrasonic guided wave arrays. First, finite element modeling was employed to simulate guided wave data acquisition by a circular array within an aluminum–epoxy bilayer structure with embedded delamination defects. Subsequently, the full waveform inversion algorithm was applied to reconstruct both regular and irregular delamination defects. Analysis results indicated the efficacy of the proposed approach in accurately identifying delamination defects of varying shapes. Furthermore, an experimental platform for guided wave delamination defect detection was established, and experiments were conducted on a steel–cement bilayer structure containing an irregular delamination defect. The experimental results validated the exceptional imaging precision of our proposed technique for identifying delamination defects in multi-layered boards. In summary, the proposed method can accurately determine both the positions and sizes of defects with higher detection efficiency than traditional pulse-echo delamination detection methods.

## 1. Introduction

Adhesive structures comprise assemblies formed by bonding metals to metals, metals to non-metals, or non-metals to non-metals using adhesives. These structures are renowned for their lightweight nature, cost-effectiveness, and ease of manufacture. Additionally, owing to their exceptional fatigue resistance, high-temperature tolerance, corrosion resistance, and uniform stress distribution, adhesive structures have garnered extensive utilization across critical sectors such as aviation, aerospace, automotive, and artificial satellite industries [1]. However, throughout their service life, adhesive structures are susceptible to delamination at their bonding interfaces due to factors such as adhesive processes, environmental conditions, and aging. Consequently, advancing and employing adhesive structures necessitates a dependable assessment of their integrity during research, development, and operational phases, achievable through non-destructive testing techniques [2].

Ultrasonic testing stands out as a significant non-destructive evaluation method, providing high-resolution detection of subsurface defects such as corrosion, delamination, voids, and cracks in materials [3,4,5]. Conventional ultrasonic testing typically employs bulk waves, characterized by high frequency and short wavelengths relative to the component thickness. While this approach yields high detection accuracy, it exhibits lower efficiency when dealing with large-area components. By contrast, guided wave inspection employs signals with wavelengths comparable to the thickness of the structural walls, enabling the inspection of large-area components [6]. Guided wave non-destructive testing and structural health monitoring employ guided wave array imaging technology, which facilitates rapid localization and identification of cracks, through-holes, and delamination damage in plate-like structures [7].

Various guided wave array imaging techniques are employed for delamination detection, including reverse-time migration, spatial wavenumber filter, time-reversal imaging, machine learning imaging, and tomography. Reverse-time migration and spatial wavenumber filters primarily employ reflection wave imaging, which is notably influenced by edge reflections of the specimen [8,9]. While time-reversal imaging techniques can achieve sub-wavelength super-resolution imaging, they cannot precisely evaluate sub-wavelength damage features such as location, size, and type, resulting in a lack of crucial information for effective non-destructive testing [10,11]. Machine learning imaging methods offer high accuracy; however, these methods lack interpretability of the underlying physical processes, often necessitating large amounts of training data [12]. Guided wave tomography, on the other hand, detects defects by harnessing the dispersion properties of guided waves. The technique deploys transducer arrays around the region of interest, enabling the inversion of collected ultrasonic guided wave signals and the reconstruction of areas of material debonding [13]. Existing guided wave tomography algorithms primarily include travel-time tomography [14,15], diffraction tomography [16], HARBUT tomography [17,18], and full waveform inversion tomography [19].

Compared with alternative tomography methodologies, full waveform inversion (FWI) stands out for its ability to achieve superior resolution through the comprehensive integration of waveform travel times, phases, and amplitudes, even under conditions of limited data availability. This approach facilitates multi-parameter modeling, thereby encapsulating the intricate acoustic wave dynamics inherent in predictive data simulations [20,21]. Within the domain of industrial non-destructive testing, FWI has garnered considerable attention for its efficacy in both bulk wave and guided wave detection. In the context of bulk wave detection, Nguyen et al. applied the FWI algorithm to discern the stratification within concrete bridge decks, yielding promising outcomes in both simulated and experimental settings [22]. He et al. [23] extended the application of FWI to irregularly shaped objects, showcasing its capacity to accurately reconstruct inclusions within gear assemblies based on numerical simulations. Furthermore, Rao integrated FWI with the reverse time migration (RTM) algorithm, achieving high-resolution reconstruction of void defects within multi-layered materials [24]. In guided wave detection, pioneering work by Rao et al. [25] employed FWI for the reconstruction of residual thickness in aluminum plates, leveraging both numerical simulations and experimental A0 mode wave measurements to achieve remarkable imaging resolution. The FWI method surpasses conventional tomographic techniques in terms of imaging fidelity [26]. Additionally, the utilization of FWI enabled researchers to conduct a comprehensive analysis of velocity and attenuation characteristics associated with the S0 mode, facilitating high-resolution reconstruction of corrosion defects in aluminum plates [27]. Ratassepp et al. [28] refined forward modeling techniques to elucidate wave propagation behaviors within anisotropic plates, resulting in precise reconstructions of delamination locations and extent within composite material plates.

Currently, FWI-based guided wave tomography (GWT) technology primarily targets the detection of single-layer or anisotropic materials, with relatively less research focused on detecting delamination defects in multi-layered materials. Therefore, this paper proposes a novel methodology for detecting delamination defects in double-layered boards utilizing FWI. Compared to traditional bulk wave detection methods, this significantly enhances detection efficiency. Both simulation and experimental results underscore the method’s efficacy in detecting delamination defects with high accuracy. The subsequent sections of this paper are structured as follows: Section 2 provides a succinct overview of guided wave detection principles based on FWI. Section 3 presents numerical studies validating the algorithm’s efficacy in detecting delamination defects of varying geometries within aluminum–epoxy bilayer materials. Section 4 comprises experimental validation, wherein the proposed method is applied to detect irregular delamination defects within steel–cement bilayer boards. Finally, Section 5 offers concluding remarks.

## 2. Method

### 2.1. Full Waveform Inversion

**Figure 1:**
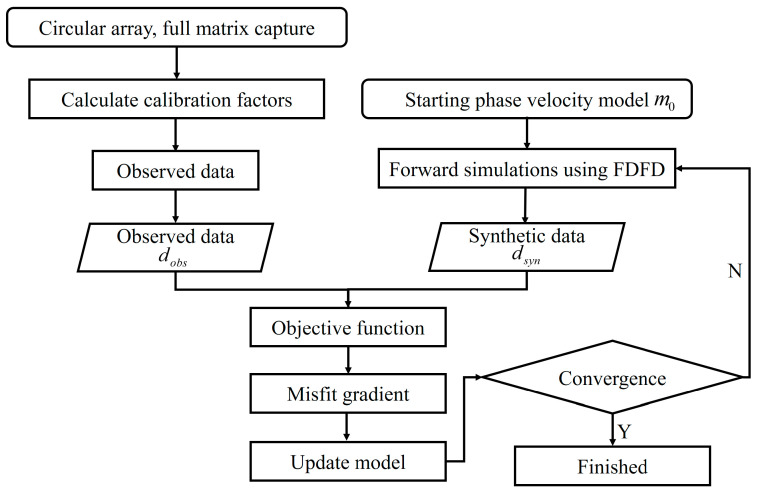
Flow diagram of the GWT algorithm based on FWI.

The full waveform inversion method comprises two key components: (1) forward simulation based on predefined model parameters and (2) calculation of the objective function gradient and parameter updates using a suitable optimization algorithm. The flow diagram of the FWI-GWT is illustrated in Figure 1.

#### 2.1.1. Forward Simulation

In the gradient computation phase of FWI, two forward wavefield simulations are necessary: (1) generating the incident wavefield based on the source and (2) computing the residual at the receiver location and utilizing it as a source to compute the back-propagated wavefield. Hence, the efficiency of gradient computation is directly linked to the efficiency of forward simulation. In guided wave detection, the propagation of guided waves in thin structures is commonly described using the constant-density acoustic wave equation. In the frequency domain, this equation can be represented by the Helmholtz equation:

(1) $$\left(\nabla^{2}+{\frac{\omega }{v^{2}}}^{2}\right)p(r,\omega )=-s(\omega )\delta (r-r_{s})$$

where $\nabla^{2}=\frac{\partial }{\partial x^{2}}+\frac{\partial }{\partial y^{2}}=\partial_{x}^{2}+\partial_{y}^{2}$ represents the Laplace operator, ω=2πf denotes the angular frequency, and f denotes frequency; v denotes the speed of wave propagation; r(x,y) denotes the position in two-dimensional space and p(r,ω) denotes the acoustic pressure field; s(ω) represents the spectrum of the source signal; $\delta (r-r_{s})$ represents the Dirac function; and $r_{s}$ denotes the position of the sound source in two-dimensional space. In this study, we employed a nine-point differencing scheme to discretize Equation (1) [29]. To minimize interference from boundary reflections, perfectly matched layers were positioned around the computational domain to absorb the reflected acoustic wave energy. Equation (1) can be represented using matrices through finite-difference discretization:

(2) Ap=s

where $A=\nabla^{2}+\frac{\omega^{2}}{v^{2}}$ is a N×N complex impedance matrix associated with material properties, frequency, boundary conditions, and discretization format; N represents the total number of grid points in the two-dimensional grid, while p and s are N×1 vectors representing the acoustic pressure field and the source function, respectively. To enhance computational efficiency, the LU decomposition [30] can be employed to solve the wavefield p:

(3) $$LU[p_{1},p_{1},\ldots ,p_{n}]=[s_{1},s_{2},\ldots ,s_{n}]$$

where L and U represent the upper triangular matrix and lower triangular matrix of the LU decomposition, respectively. This method employs the decomposed matrix A to simultaneously solve the forward problems for multiple sources, notably accelerating the computation speed.

In our inversion method, to enhance efficiency, the forward modeling process employs the acoustic wave equation to simulate the propagation of guided waves, which differs from actual guided wave propagation. Therefore, it is necessary to calibrate the measured data to reduce the differences between these two models [25]. The method of calibrating data involves calculating correction factors based on Equation (4) and multiplying the measured data or finite element simulation data by these correction factors to reduce discrepancies. The calibration factor *Q* can be represented as follows:

(4) $$Q=\frac{fft(\psi_{0})}{fft(d_{obs,0})}$$

where $d_{obs,0}$ represents the time-domain signal unaffected by defect scattering obtained from finite element simulations or experimental measurements, $\psi_{0}$ represents the time-domain signal in a homogeneous medium simulated using finite difference methods, and fft() denotes the fast Fourier transform.

#### 2.1.2. Inversion Theory

For a specific single-frequency component, the frequency domain L2 objective function can be expressed as follows:

(5) $$E(v)=\frac{1}{2}{\sum_{x_{s}}\sum_{x_{r}}\left[Rd_{syn}(\omega ,x,x_{s};v)-d_{obs}(\omega ,x_{r},x_{s})\right]}^{2}$$

where $d_{syn}(\omega ,x,x_{s};v)$ denotes the synthetic data related to the model parameters v, $d_{obs}(\omega ,x_{r},x_{s})$ are the observed data, and R denotes the operator associated with the receiver positions.

To circumvent the computation and storage challenges posed by the enormous Frechet derivative matrix, this study employed the adjoint-state method [31] to calculate the gradient of the objective function with respect to velocity. The expression for the gradient is as follows:

(6) $$g(v)=\frac{\delta E(v)}{\delta v}=-\frac{2\omega^{2}}{v^{3}}\sum_{x_{s}}d_{syn}B^{*}[R^{*}(Rd_{syn}-d_{obs})]$$

where B denotes the forward operator and holds that $B=A^{-1}$. Assuming $u=B^{*}[R^{*}(Ru-d_{obs})]$,

(7) $$g(v)=-\frac{2\omega^{2}}{v^{3}}\sum_{x_{s}}dp$$

The gradient is computed based on the results derived from the incident wavefield d and the back-propagated wavefield p.

Given the gradient expression, the process of updating the model parameters can be formulated as follows:

(8) $$v^{\left(k+1\right)}=v^{\left(k\right)}-\alpha g^{\left(k\right)}$$

where α represents the optimization step length, $g^{\left(k\right)}$ denotes the gradient value at the *k*-th iteration, and $v^{\left(k\right)}$ represents the model parameters at the *k*-th iteration.

Considering only first-order derivatives, the corresponding technique is the steepest descent method. However, this method exhibits slow convergence speed, thereby limiting its applicability to large-scale problems. By contrast, the Newton algorithm possesses second-order convergence properties and high solution accuracy. The iterative process for updating the model parameters using the Newton algorithm can be expressed as follows:

(9) $$v^{\left(k+1\right)}=v^{\left(k\right)}-\alpha H^{-1(k)}g^{\left(k\right)}$$

where $H^{-1(k)}$ represents the inverse of the Hessian matrix. Nevertheless, computing the Hessian matrix imposes significant demands on the computational power and storage space of computers. Therefore, the limited-memory Broyden–Fletcher–Goldfarb–Shanno (L-BFGS) method [32] is employed for updating the model. This method approximates the Hessian matrix by disregarding complex terms, leading to faster convergence compared to the first-order steepest descent method and a notably lighter computational burden compared to the second-order Newton method.

### 2.2. Regularization Methods

In the iterative imaging process, Gaussian filtering is a widely utilized technique to mitigate the effects of noise and artifacts, thereby enhancing imaging precision. For the FWI algorithm based on the elastic model, the resolution typically ranges between 1.5 and 2λ. Based on empirical knowledge, in this study, the standard deviation of the Gaussian filter is set to λ/2, where λ represents the wavelength.

In addition to the aforementioned methods, we also employ variable relaxation regularization and threshold regularization methods [33]. Threshold regularization relies on the actual phase velocity dispersion curve, where unreasonable reconstructed velocity values are adjusted to match the background velocity. Conversely, variable relaxation regularization is inspired by Tikhonov regularization. It regulates the amplification of spatial fluctuations in each iteration, stemming from noise and sensor limitations, thereby facilitating the precise reconstruction of localized defects above the noise threshold. Denoted as $v_{ij}$ before regularization and $v_{ij}^{\prime }$ after regularization, variable relaxation regularization can be defined as follows:

(10) $$v_{ij}^{\prime }=\left\{\begin{array}{cc} \tilde{v}+\frac{v_{ij}-\tilde{v}}{{\left(1+\frac{1}{z_{ij}^{2}}\right)}^{\frac{\alpha }{2}}} & \;0\text{<}\left|v_{ij}-\tilde{v}\right|\text{<}\gamma \beta \tilde{v}\;\\ v_{ij} & \;\mathrm{otherwise}\; \end{array}\right.\;z_{ij}=\left\{\begin{array}{cc} \frac{\left|v_{ij}-\tilde{v}\right|}{\beta \tilde{d}} & \left|v_{ij}-\tilde{v}\right|<\beta \tilde{v}\\ \frac{\left|v_{ij}-\tilde{v}\right|}{0.5\beta \left[1-\cos(\pi \frac{\left|v_{ij}-\tilde{v}\right|-\gamma \beta \tilde{v}}{(1-\gamma )\beta \tilde{v}})\right]} & \;\beta \tilde{v}\leq \left|v_{ij}-\tilde{v}\right|<\gamma \beta \tilde{v} \end{array}\right.$$

where $\tilde{v}$ represents the reference velocity for regularization, typically aligned with the velocity of the homogeneous model. $\beta \in \left[0,\;1\right]$ denotes a value contingent upon data noise, with larger values resulting in diminished background noise post-regularization. $\gamma \in \left[1,+\infty \right)$ stands for the relaxation constant, and the product of γ and β determines the extent of regularization on velocity; a larger product yields a broader range of regularization. $\alpha \in {\mathbb{R}}_{+}$ constitutes a constant that defines the strength of regularization; higher values translate to fewer artifacts in the reconstructed velocity map, albeit with larger reconstruction errors. This study set α=2, β=0.1, and γ=3.

### 2.3. Error Analysis

The root mean square error (RMSE) is used to assess the reconstruction accuracy. The expression of the RMSE is as follows:

(11) $$RMSE=\sqrt{\frac{{\left\|\tilde{v}-v\right\|}_{2}^{2}}{N}}$$

where $\tilde{v}$ is the true phase velocity, v is the velocity reconstructed by the FWI, and N is the total number of pixels. A smaller RMSE corresponds to a more accurate reconstruction of the defects.

## 3. Numerical Test

**Figure 2:**
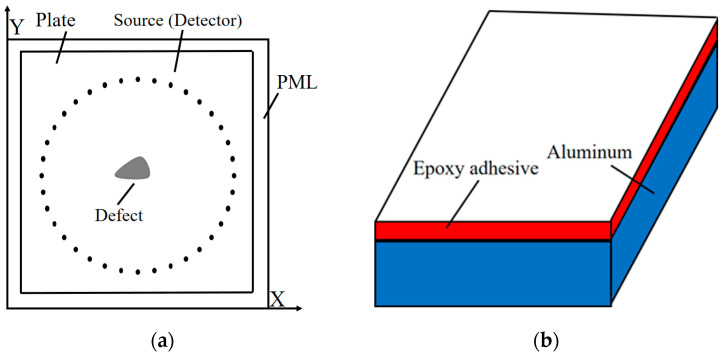
Finite element simulation model for debonding detection of aluminum–epoxy layered plates. (**a**) Geometrical schematic for marking the transducer position. (**b**) Aluminum–epoxy adhesive bonding model.

Figure 2 depicts the finite element simulation model of a double-layered bonded structure comprising primarily aluminum and epoxy. To mitigate boundary-induced reflection waves, a 30 mm absorbing boundary is extended outward from a 400 mm × 400 mm research area. Transducers are evenly distributed along a 200 mm radius circle centered at the geometric center of the bonded plate. The irregular gray area represents the debonding defect.

Table 1 provides the material parameters of the aluminum–epoxy laminate. Based on these parameters, we computed the dispersion curve. Given that the A0 mode is an asymmetric mode and is more readily excited in practical inspection processes, we have opted for the A0 mode for debonding defect detection in this study.

**Figure 3:**
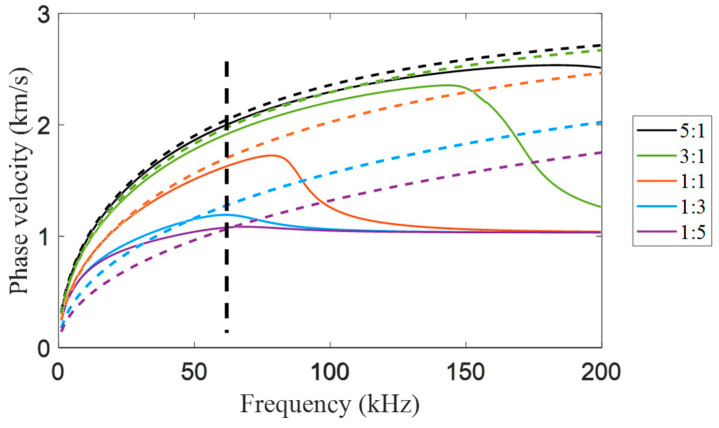
Dispersion curve of A0 mode phase velocity under different thickness ratios of aluminum–epoxy adhesive laminates. The position of the black dashed line indicates a frequency of 70 kHz.

Assuming the total thickness of the aluminum–epoxy bilayered plates is 12 mm, the solid lines of different colors in Figure 3 show the A0 mode phase velocity dispersion curves of the aluminum–epoxy layer for different thickness ratios. In the theoretical simulation part of this study, we represent the debonding of the aluminum–epoxy bilayered plates by the complete disappearance of the epoxy layer. Therefore, we have also plotted the dispersion curves of aluminum at the corresponding thickness, as shown by the dashed lines of different colors in the figure. From these dispersion curves, it is evident that for frequencies greater than 70 kHz (black dashed line), complete debonding (disappearance of epoxy) in aluminum–epoxy bilayered plates leads to an increase in the phase velocity of the A0 mode within the debonding region. The full waveform inversion algorithm relies on capturing these changes in phase velocity to detect the debonding region. Additionally, at higher frequencies, the phase velocity differences induced by debonding become more pronounced. However, simultaneously, more modes emerge, increasing the complexity of the signal processing. Therefore, selecting appropriate frequencies is crucial in practical inspection processes.

For the debonding detection issue in multi-layered plates, it is essential not only to consider the impact of phase velocity changes induced by debonding on reconstruction accuracy but also to account for the influence of transducer arrangement. Below, we will separately verify the effects of these two factors.

### 3.1. The Effect of the Degree of Phase Velocity Variation on Detection Accuracy

**Figure 4:**
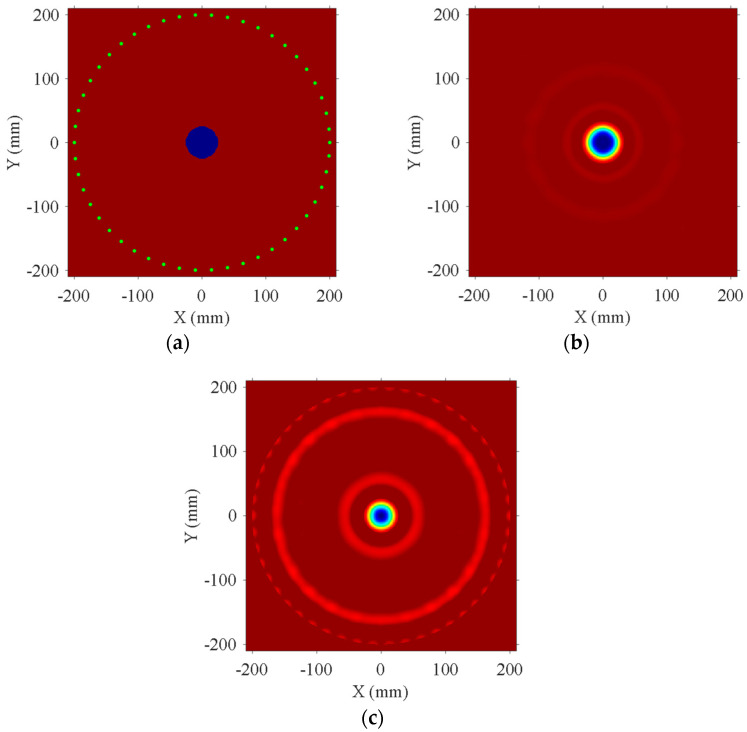
Reconstruction results of aluminum and epoxy adhesive at different thickness ratios. (**a**) Original model; (**b**) 5:1; (**c**) 1:3.

Figure 4a presents a model diagram of a circular debonding defect located at the center of an aluminum–epoxy adhesive bilayer structure. The blue-filled area in the figure indicates the circular debonding region with a diameter of 50 mm, which is made of aluminum. This area exhibits a faster phase velocity compared to the aluminum–epoxy structure. According to Huthwaite [17], the maximum transducer spacing is recommended to be less than half a wavelength. However, deploying an excessive number of transducers in practical experiments is not feasible. Consequently, transducer spacing is usually set within one wavelength [26]. Fifty transducers, centered at a frequency of 80 kHz, are evenly distributed along the circumference of a circle with a radius of 200 mm on one side of the aluminum plate. At this spacing, the distance between transducers is less than one wavelength for the A0 mode. Using the phase velocity of the A0 mode under well-bonded conditions (aluminum–epoxy) as the initial model, Figure 4b,c show the debonding defect images reconstructed using the FWI algorithm for different thickness ratios of aluminum and epoxy. In comparison, Figure 4c exhibits a higher degree of artifacts, primarily because the phase velocity change between well-bonded and debonded conditions at this thickness ratio is only 3 m/s. Table 2 presents the RMSE for the two thickness ratios, showing that the reconstruction accuracy is higher when the thickness ratio is 5:1. Therefore, in practical detection processes, more significant phase velocity changes are advantageous for improving detection accuracy.

### 3.2. The Effect of Transducer Installation Position on Detection Accuracy

**Figure 5:**
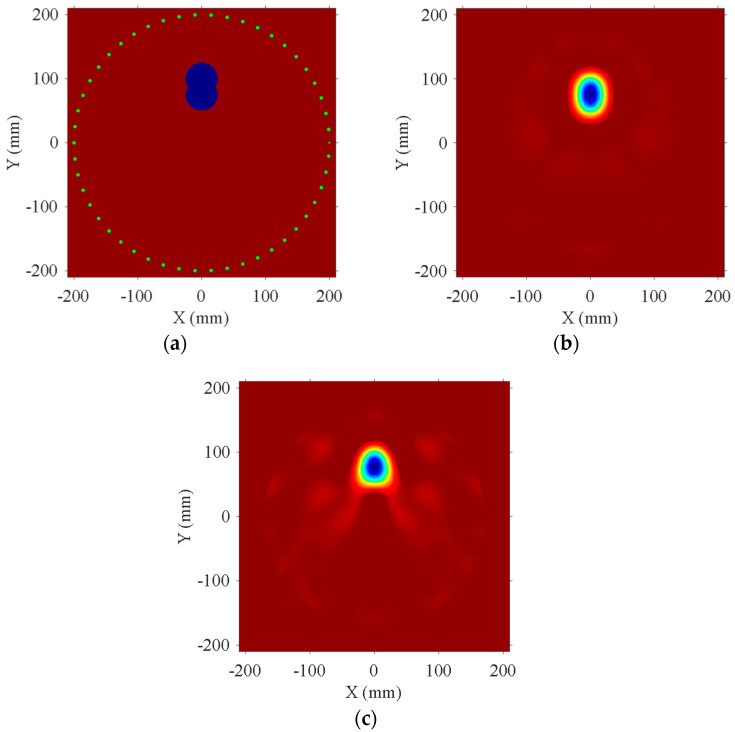
Reconstruction results of different transducer installation positions. (**a**) Original model. (**b**) Transducer mounted on aluminum plate surface. (**c**) Transducer mounted on epoxy adhesive surface.

**Figure 6:**
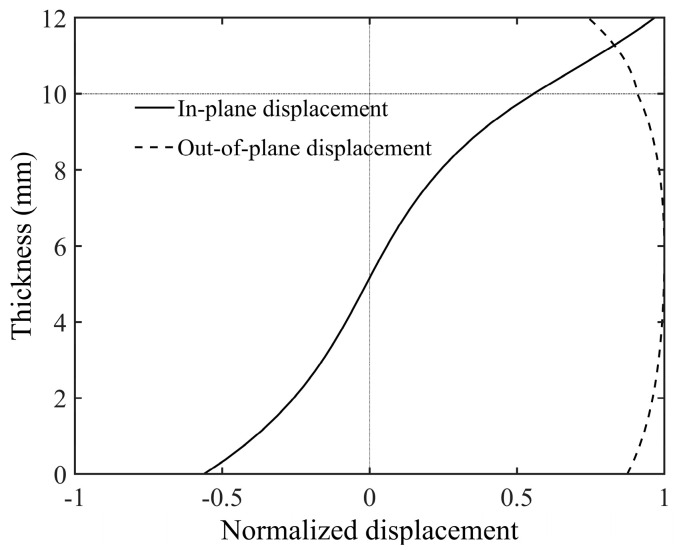
Displacement wave structure of the A0 mode at 80 kHz in aluminum–epoxy adhesive laminate.

Considering that debonding defects in practical situations may have various irregular shapes, an irregular debonding area was set up on the model of a bilayer plate with an aluminum–epoxy thickness ratio of 5:1, as shown in Figure 5a. This irregular shape consists of two circular regions with a radius of 25 mm each. Similar to Section 3.1, the blue-filled area in the figure indicates the debonding region, which is made of aluminum. This area exhibits a faster phase velocity compared to the aluminum–epoxy structure. Fifty transducers, with a center frequency of 80 kHz, are evenly spaced along a circumference with a radius of 200 mm. The acoustic source is loaded in the normal direction. The reconstruction result from the transducer array placed on the aluminum plate surface is shown in Figure 5b, and the reconstruction result from the transducer array placed on the epoxy surface is shown in Figure 5c. Table 3 shows the RMSE of the reconstruction results with transducers installed at different positions. The imaging results indicate that the debonding defect location can be accurately reconstructed regardless of which side of the plate the transducers are installed on. Additionally, applying the normal displacement source on the aluminum plate surface achieves higher imaging resolution. To analyze the reason for this, we plotted the displacement wave structure characteristics of the A0 mode at 80 kHz, as shown in Figure 6. The solid line represents in-plane displacement, while the dashed line represents out-of-plane displacement. From the figure, it can be seen that on the aluminum plate surface, the A0 mode primarily exhibits out-of-plane displacement vibration. Therefore, installing the transducers on the aluminum plate surface is more advantageous for exciting the A0 mode, thereby achieving higher detection accuracy.

## 4. Experimental Test

**Figure 7:**
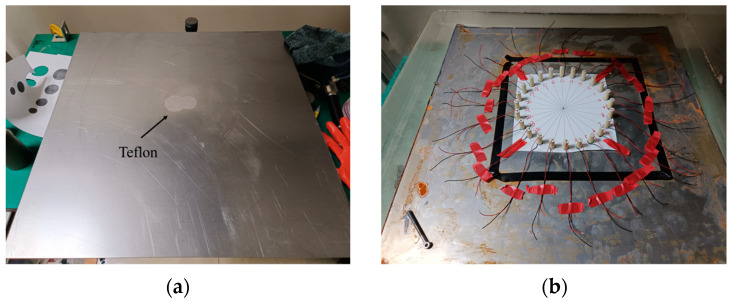
Guided wave detection experiment photo of a steel–cement bilayer plate. (**a**) Irregularly shaped PTFE film adhered to a 2 mm thick steel plate. (**b**) Twenty-four piezoelectric transducers mounted on the steel–cement bilayer plate.

To further validate the feasibility of FWI-guided wave tomography in practical inspection processes, corresponding experiments on guided wave detection of the bilayer board were conducted. As illustrated in Figure 7a, a PTFE film was affixed to the interface of the steel–cement bilayer plate to simulate an area with poor adhesion. Subsequently, cement was poured onto the steel plate’s surface and allowed to settle for 48 h, resulting in the steel–cement bilayer structure depicted in Figure 7b. This structure comprises a 2 mm thick cold-rolled steel plate and a 10 mm thick cement layer. The longitudinal and shear wave velocities, as well as the densities of steel and cement, were measured and are shown in Table 4.

### 4.1. Pulse-Echo Method

**Figure 8:**
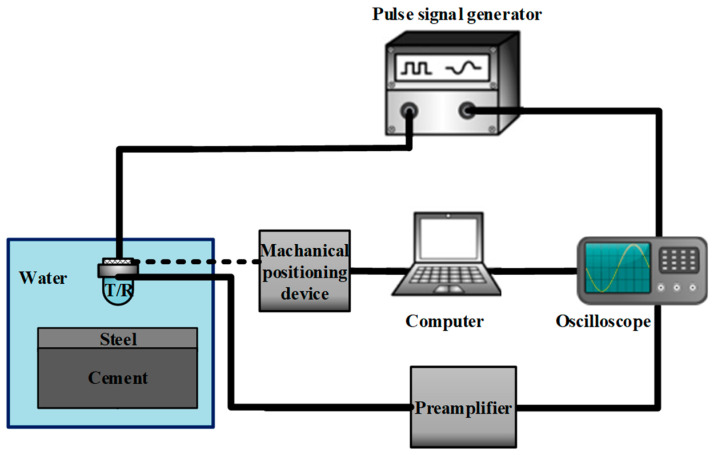
Experimental equipment and measurement principles for pulse-echo debonding defect detection.

**Figure 9:**
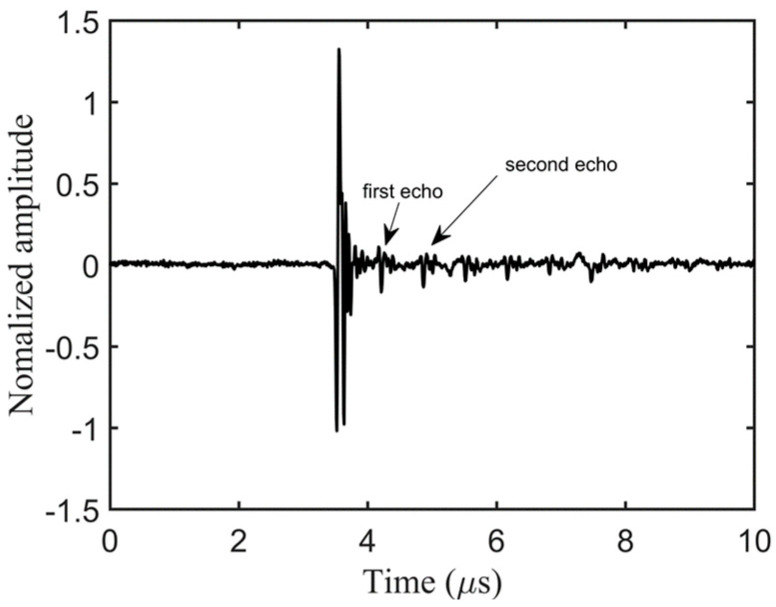
Steel–cement multiple wave measurement signals.

**Figure 10:**
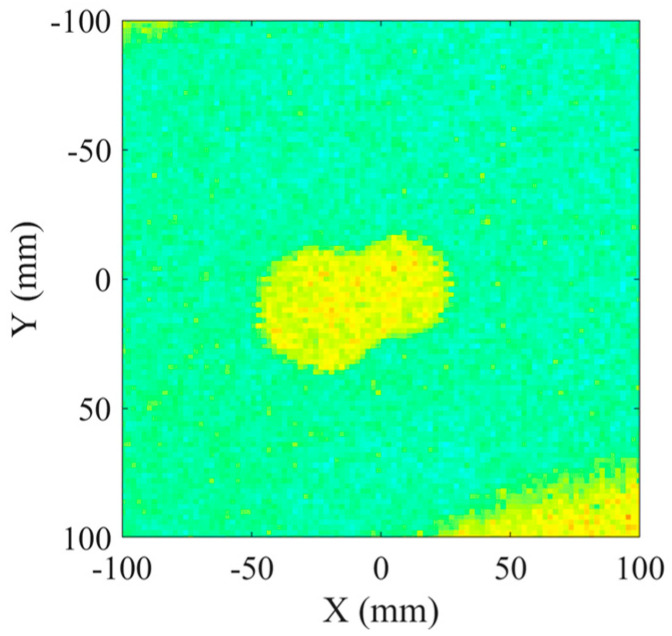
Reconstruction results of the pulse-echo method.

To assess the bonding quality, we initially employed the conventional pulse-echo method for inspecting the bonding condition of the steel–cement bilayer plate. Figure 8 illustrates the experimental setup for pulse-echo debonding detection. The bilayer plate in the lower left corner represents the test specimen, with “T” denoting the emitter and “R” indicating the receiver. During measurements, the emitter transmits ultrasonic waves vertically toward the plate. A mechanical positioning device is employed to comprehensively inspect the specimen using the “S”-shaped water immersion scanning method. The experiment utilized an 5072PR (Olympus, Tokyo, Japan) pulse signal generator as the excitation device, which stimulated the emitter. Additionally, the emitter served as the receiver to collect the signals. The collected signals passed through a preamplifier, were displayed on an oscilloscope, and were stored in real time on a computer. Figure 9 shows multiple echo measurement signals obtained at one of the positions on the plate [35]. The first and largest peak represents the echo signal from the top surface of the plate and does not contain any information about defects within the plate. The first and second echoes indicated by arrows are the signals used to assess the bonding quality. To determine the presence of debonded areas, one only needs to calculate the ratio of the maximum peak of the first echo to the maximum peak of the second echo. Figure 10 displays the imaging results obtained after multiple-wave amplitude processing of the collected array data. The yellow area in the image indicated poor bonding quality, while the green area indicated good bonding. The image clearly revealed an “8”-shaped debonding defect in the middle caused by the PTFE film, confirming the experiment’s feasibility. However, despite offering high imaging accuracy, traditional ultrasonic scanning is relatively slow. Therefore, we evaluated the bonding quality using the guided wave tomography technique based on full waveform inversion.

### 4.2. FWI Method

**Figure 11:**
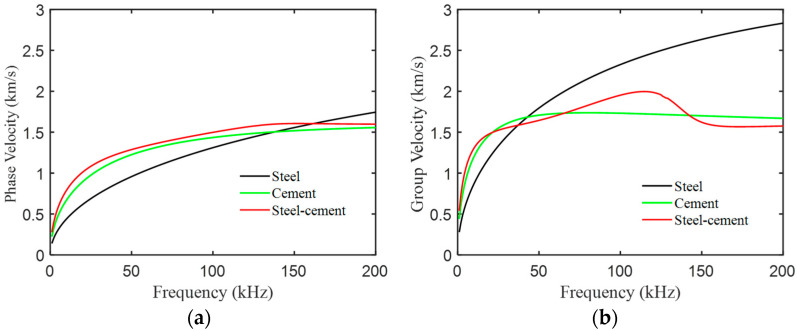
Dispersion curve of A0 mode waves in steel–cement laminated plate. (**a**) Phase velocity. (**b**) Group velocity.

**Figure 12:**
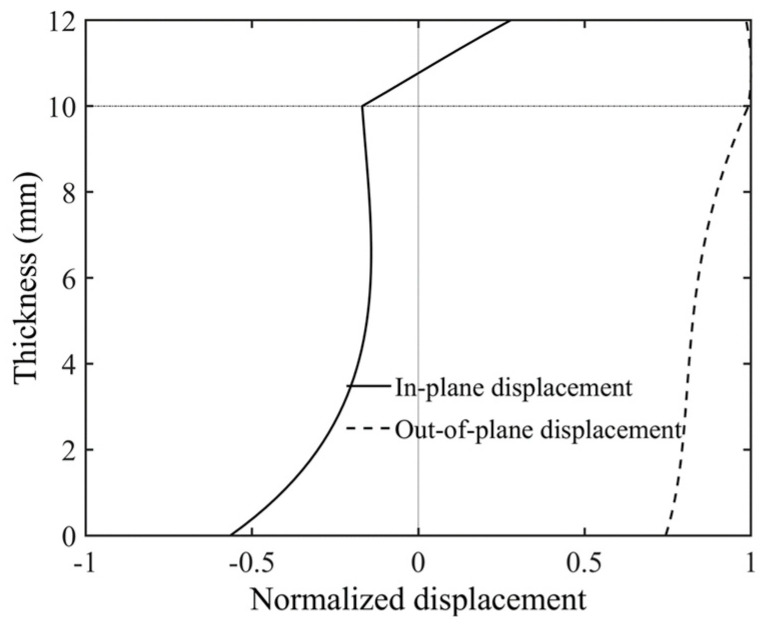
Displacement wave structure of the A0 mode at 50 kHz in a steel–cement laminate.

**Figure 13:**
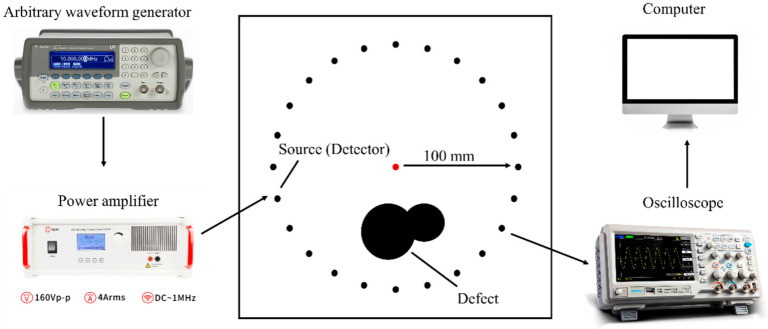
Experimental setup.

Based on longitudinal and transverse wave velocities from Table 4, we plotted the dispersion curves of the A0 mode wave in the bilayer board under various bonding qualities, as illustrated in Figure 11. Given the smoother surface of the steel plate, we mounted the transducers on it. Analysis of the dispersion curves revealed that the phase velocity difference between the steel and steel–cement bilayer plates initially increased and then decreased with increasing frequency within the frequency-thickness product range of 0-100 kHz×mm. Therefore, we selected a five-cycle Hanning window-modulated cosine with a center frequency of 50 kHz as the excitation signal. Figure 12 displays the displacement wave structure curve of the A0 mode wave in the steel–cement bilayer plate at 50 kHz, demonstrating predominantly out-of-plane displacement on the steel plate side. Therefore, we arranged 24 d31-mode piezoelectric transducers (Baoding Hongsheng Ceramic Inc., Baoding, China) at the geometric center of the steel side of the steel–cement bilayer plate, as illustrated in Figure 7b. These transducers were evenly spaced along a circumference with a radius of 100 mm and were directly attached to the steel plate surface using AB glue. Each d31 piezoelectric sensor had a diameter of 10 mm and a length of 26 mm. Figure 13 illustrates the experimental setup for array data acquisition, comprising a computer, power amplifier, signal generator, oscilloscope, ultrasonic transducers, and the stee–cement bilayer board. The experiment employed a power amplifier to amplify the signal from the signal generator, serving as the input to the emitter. Subsequently, the received signal on the receiver was observed on an oscilloscope, which was connected to a computer for real-time storage of the acquired data.

**Figure 14:**
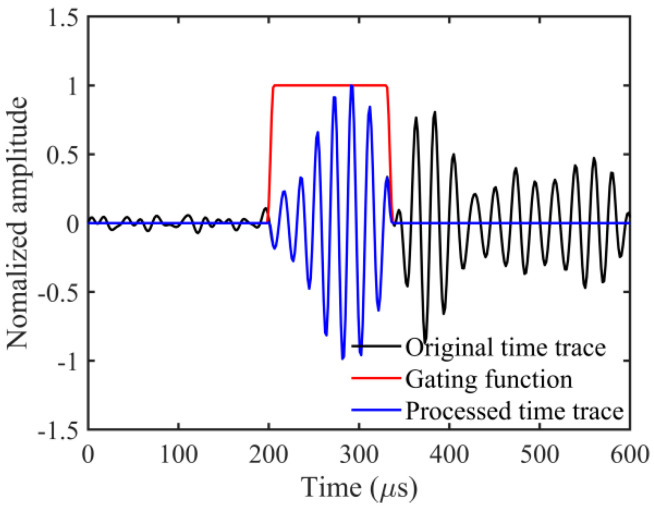
Waveform acquired from an emitter–receiver pair.

During the signal acquisition process, noise often accompanies the data. Interference signals can distort measurements and must be mitigated. To address this, a bandpass filter tailored to the transducer’s bandwidth is applied. In practical settings, unlike simulated scenarios, measurements are influenced by reflected waves from the plate’s edges. These reflections, devoid of defect-related information, are filtered out. In this study, the method outlined in [36] was employed to extract the A0 mode. The method involved applying a Tukey window to the original signals based on the group velocity dispersion curve, as shown in Figure 11b. Figure 14 displays the waveform captured by a single emitter–receiver pair. The black solid line represents the normalized original waveform, the red solid line depicts the Tukey window function, and the blue solid line illustrates the extracted A0 mode. After the Fourier transformation of the A0 mode data, the frequency-domain FWI method was employed for defect imaging.

**Figure 15:**
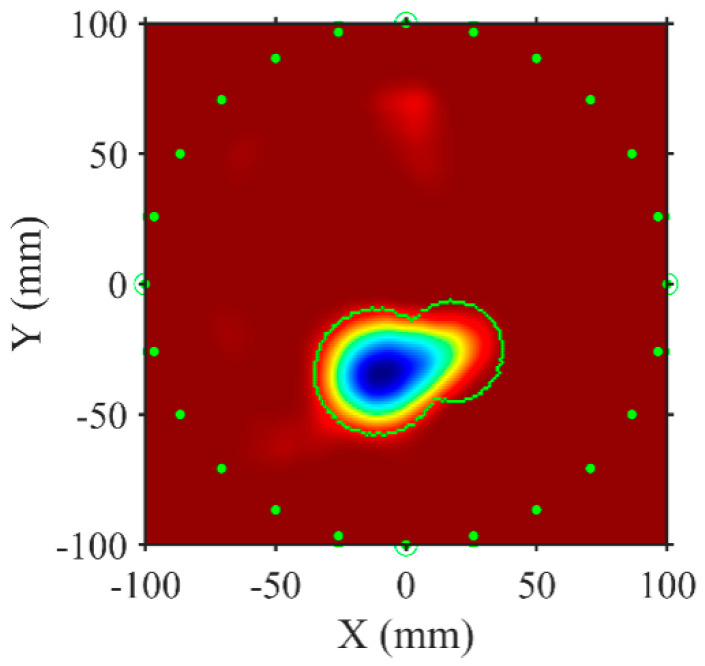
Reconstruction results of delamination defects in steel–cement laminated plate. Green dots mark the positions of the sensors. The Green dashed line represents the contour of the actual position of the PTFE film.

Figure 15 presents the reconstructed location of the delamination defect at the highest frequency obtained via the FWI algorithm. The blue area delineates the reconstructed delamination region, while the red area denotes the uniformly bonded region. The green dots mark the transducers’ positions, and the green dashed lines indicate the original delamination region’s extent. Despite minor shape distortions and artifacts, likely due to uneven cement thickness, the position of the delamination area is accurately reconstructed. These results confirm the effectiveness of the proposed method in reconstructing the delamination area’s shape and position. Compared with traditional pulse-echo delamination detection methods, the FWI-based guided wave detection method offers expedited detection.

## 5. Conclusions

This study established a method for detecting delamination defects in bilayer structures using ultrasonic guided wave arrays based on the FWI algorithm. Numerical investigations demonstrated that FWI-based guided wave debonding defect detection effectively captures changes in phase velocity caused by defects, enabling accurate defect identification. Regardless of regular or irregular patterns in the delamination area, the proposed method reliably reconstructs the approximate defect position. Delamination detection in steel–cement bilayer plates revealed that the new detection approach, compared to traditional pulse-echo methods, enhanced detection efficiency.

The research in this study focuses on the detection of delamination defects in multi-layer plates. When the thickness of the pipe wall is much smaller than the pipe diameter, and the guided wave wavelength is smaller than or comparable to the thickness of the pipe wall, the propagation characteristics of the guided wave in the plate are similar to those in the pipe wall [37]. Currently, there is relatively little research on delamination defect detection in multi-layer pipes in aerospace, petrochemical, and other fields. Corresponding work will be conducted in the future.

## Figures and Tables

**Table 1:** Material parameters of aluminum–epoxy laminated plate [34].

Material	*ρ* (kg/m^3^)	*C_T_* (m/s)	*C_S_* (m/s)
Aluminum	2700	6364	3170
Epoxy adhesive	1170	2610	1102

**Table 2:** RMSE of reconstruction results for aluminum and epoxy adhesive at different thickness ratios.

Thickness Ratio	RMSE (m/s)
5:1	1.80
1:3	2.97

**Table 3:** RMSE of reconstruction results for different transducer installation positions.

Transducer Installation Positions	RMSE (m/s)
Aluminum plate surface	1.92
Epoxy adhesive surface	2.21

**Table 4:** Material parameters of the steel–cement laminated plate.

Material	*ρ* (kg/m^3^)	*C_T_* (m/s)	*C_S_* (m/s)
Steel	7850	5890	3240
Cement	1865	2930	1741

## Data Availability

The data underlying the results presented in this paper are not publicly available at this time but may be obtained from the authors upon reasonable request.

## References

[1] Guyott C.C.H., Cawley P., Adams R.D. (1986). The non-destructive testing of adhesively bonded structure: A review. J. Adhes..

[2] Cawley P. (1992). Ultrasonic measurements for the quantitative NDE of adhesive joints-potential and challenges. Proceedings of the IEEE 1992 Ultrasonics Symposium.

[3] Honarvar F., Varvani-Farahani A. (2020). A review of ultrasonic testing applications in additive manufacturing: Defect evaluation, material characterization, and process control. Ultrasonics.

[4] Fang Y., Lin L., Feng H., Lu Z., Emms G.W. (2017). Review of the use of air-coupled ultrasonic technologies for nondestructive testing of wood and wood products. Comput. Electron. Agric..

[5] Szilard J. (1982). Ultrasonic Testing: Non-Conventional Testing Techniques.

[6] Zhao X., Royer R.L., Owens S.E., Rose J.L. (2011). Ultrasonic Lamb wave tomography in structural health monitoring. Smart Mater. Struct..

[7] Liu Z., Zhong X., Xie M., Liu X., He C., Wu B. (2017). Damage imaging in composite plate by using double-turn coil omnidirectional shear-horizontal wave magnetostrictive patch transducer array. Adv. Compos. Mater..

[8] He J., Yuan F.G. (2015). Damage identification for composite structures using a cross-correlation reverse-time migration technique. Struct. Health Monit..

[9] Qiu L., Liu B., Yuan S., Su Z., Ren Y. (2016). A scanning spatial-wavenumber filter and PZT 2-D cruciform array based on-line damage imaging method of composite structure. Sens. Actuators A Phys..

[10] Zhao G., Zhang D., Zhang L., Wang B. (2018). Detection of defects in reinforced concrete structures using ultrasonic nondestructive evaluation with piezoceramic transducers and the time reversal method. Sensors.

[11] Wang J., Shen Y. (2019). An enhanced Lamb wave virtual time reversal technique for damage detection with transducer transfer function compensation. Smart Mater. Struct..

[12] Tong J., Lin M., Wang X., Li J., Ren J., Liang L., Liu Y. (2022). Deep learning inversion with supervision: A rapid and cascaded imaging technique. Ultrasonics.

[13] Qian Z., Wang B., Zhang Y., Wang S., Li P., Qian Z., Kuznetsova I. (2023). Guided Wave Tomography of Surface Defects Based on the Method of Moments. Adv. Theory Simul..

[14] McKeon J.C.P., Hinders M.K. (1999). Parallel projection and crosshole Lamb wave contact scanning tomography. J. Acoust. Soc. Am..

[15] Malyarenko E.V., Hinders M.K. (2000). Fan beam and double crosshole Lamb wave tomography for mapping flaws in aging aircraft structures. J. Acoust. Soc. Am..

[16] Belanger P., Cawley P., Simonetti F. (2010). Guided wave diffraction tomography within the born approximation. IEEE Trans. Ultrason. Ferroelectr. Freq. Control.

[17] Huthwaite P. (2014). Evaluation of inversion approaches for guided wave thickness mapping. Proc. R. Soc. A Math. Phys. Eng. Sci..

[18] Huthwaite P., Simonetti F. (2011). High-resolution imaging without iteration: A fast and robust method for breast ultrasound tomography. J. Acoust. Soc. Am..

[19] Virieux J., Operto S. (2009). An overview of full-waveform inversion in exploration geophysics. Geophysics.

[20] Bernard S., Monteiller V., Komatitsch D., Lasaygues P.L. (2017). Ultrasonic computed tomography based on full-waveform inversion for bone quantitative imaging. Phys. Med. Biol..

[21] Pérez-Liva M., Herraiz J.L., Udías J.M., Miller E., Cox B.T., Treeby B.E. (2017). Time domain reconstruction of sound speed and attenuation in ultrasound computed tomography using full wave inversion. J. Acoust. Soc. Am..

[22] Nguyen T.D., Tran K.T., Gucunski N. (2017). Detection of bridge-deck delamination using full ultrasonic waveform tomography. J. Infrastruct. Syst..

[23] He J., Rao J., Fleming J.D., Gharti H.N., Nguyen L.T., Morrison G. (2021). Numerical ultrasonic full waveform inversion (FWI) for complex structures in coupled 2D solid/fluid media. Smart Mater. Struct..

[24] Rao J., Sing S.L., Lim J.C.W., Yeong W.Y., Yang J., Fan Z., Hazell P. (2022). Detection and characterisation of defects in directed energy deposited multi-material components using full waveform inversion and reverse time migration. Virtual Phys. Prototyp..

[25] Rao J., Ratassepp M., Fan Z. (2016). Guided wave tomography based on full waveform inversion. IEEE Trans. Ultrason. Ferroelectr. Freq. Control.

[26] Rao J., Ratassepp M., Fan Z. (2017). Investigation of the reconstruction accuracy of guided wave tomography using full waveform inversion. J. Sound Vib..

[27] Rao J., Ratassepp M., Fan Z. (2017). Quantification of thickness loss in a liquid-loaded plate using ultrasonic guided wave tomography. Smart Mater. Struct..

[28] Ratassepp M., Rao J., Yu X., Fan Z. (2021). Modeling the Effect of Anisotropy in Ultrasonic-Guided Wave Tomography. IEEE Trans. Ultrason. Ferroelectr. Freq. Control.

[29] Hustedt B., Operto S., Virieux J. (2004). Mixed-grid and staggered-grid finite-difference methods for frequency-domain acoustic wave modelling. Geophys. J. Int..

[30] Zhang Y., Sun Q. (2007). Symbol LU decomposition method of large scale sparse linear equations. Comput. Eng. Appl..

[31] Plessix R. (2006). A review of the adjoint-state method for computing the gradient of a functional with geophysical applications. Geophys. J. Int..

[32] Guitton A., Symes W.W. (2003). Robust inversion of seismic data using the Huber norm. Geophysics.

[33] Druet T., Tastet J.L., Chapuis B., Moulin E. (2019). Autocalibration method for guided wave tomography with undersampled data. Wave Motion.

[34] Zheng J. (2011). Research on Baseline Free Lamb Wave Damage Detection Method Based on Time Reversal. Master’s Thesis.

[35] Zhang Y.Y., Zhang P.Y., Yang R.R., Wen Y.T., Li Z.L. (2021). Recognition Method of Bonding Defects between Component Layers Based on Ultrasonic Testing. Meas. Control Technol..

[36] Xu W., Yuan M., Xuan W., Ji X., Chen Y. (2021). Quantitative Inspection of Complex-Shaped Parts Based on Ice-Coupled Ultrasonic Full Waveform Inversion Technology. Appl. Sci..

[37] Li J., Rose J.L. (2006). Natural beam focusing of non-axisymmetric guided waves in large-diameter pipes. Ultrasonics.

